# Semantic segmentation of synchrotron tomography of multiphase Al-Si alloys using a convolutional neural network with a pixel-wise weighted loss function

**DOI:** 10.1038/s41598-019-56008-7

**Published:** 2019-12-23

**Authors:** Tobias Strohmann, Katrin Bugelnig, Eric Breitbarth, Fabian Wilde, Thomas Steffens, Holger Germann, Guillermo Requena

**Affiliations:** 1 German Aerospace Center (DLR), Institute of Materials Research, Linder Hoehe, 51147 Cologne, Germany; 2 Helmholtz-Zentrum Geesthacht, Zentrum für Material- und Küstenforschung GmbH, Max-Planck-Straße 1, 21502 Geesthacht, Germany; 3 KS Kolbenschmidt GmbH, Karl-Schmidt-Straße, 74172 Neckarsulm, Germany; 4 Metallic Structures and Materials Systems for Aerospace Engineering, RWTH Aachen University, 52062 Aachen, Germany

**Keywords:** Engineering, Materials science, Mathematics and computing

## Abstract

Human-based segmentation of tomographic images can be a tedious time-consuming task. Deep learning algorithms and, particularly, convolutional neural networks have become state of the art techniques for pattern recognition in digital images that can replace human-based image segmentation. However, their use in materials science is beginning to be explored and their application needs to be adapted to the specific needs of this field. In the present work, a convolutional neural network is trained to segment the microstructural components of an Al-Si cast alloy imaged using synchrotron X-ray tomography. A pixel-wise weighted error function is implemented to account for microstructural features which are hard to identify in the tomographs and that play a relevant role for the correct description of the 3D architecture of the alloy investigated. The results show that the total operation time for the segmentation using the trained convolutional neural network was reduced to <1% of the time needed with human-based segmentation.

## Introduction

Synchrotron tomography is a state of the art characterization method to analyse quantitatively and three-dimensionally the microstructure of materials^1^. While tomography is per se a technique that involves handling large amounts of data, the continuously increasing brilliance of synchrotron sources combined with the use of fast imaging detectors is leading to data acquisition rates that are posing unprecedented challenges to materials scientists^2^. Particularly, the process of segmentation, i.e. the digital separation of the microstructural constituents contained in the images, can act as a bottleneck that slows down the analysis of data before any scientific interpretation of the investigated phenomena can be undertaken.

Image segmentation usually involves developing a procedure that combines the application of several image enhancing filters before phases can be separated by grey value thresholding either automatically (e.g. using local or global thresholds), manually or semi-manually^3^. Although this methodology works well in many cases, as it is demonstrated by the increasing use of 3D microstructural analysis in materials science^1^, it is imperative to come up with new methodologies that permit fast and accurate 3D imaging segmentation, especially for complex multiphase microstructures.

Deep learning algorithms and particularly convolutional neural networks (CNN) have become state of the art techniques for pattern recognition in all kinds of digital images^4,5^. While their use is extended in some disciplines such as earth observation or medicine^6,7^, their application in image analysis in materials science is beginning to see the light (e.g.^8–11^). In the present work, we explore the segmentation of 3D synchrotron X-ray tomography data of a multiphase Al-Si cast alloy using a CNN. The microstructure of this type of alloys consists of Si and various aluminide types embedded in an age-hardenable α-Al matrix^12^. The 3D architecture, connectivity and contiguity of the phases play a decisive role for understanding the thermo-mechanical and damage behaviour of these alloys (e.g.^13–15^). However, obtaining an accurate 3D segmentation of all the microstructural constituents in these alloys is a very complex and time consuming task owing to the fact that Si can only be revealed by phase contrast owing to the similar X-ray attenuation with respect to Al^16^. Thus, we present here the training of a CNN with a so-called U-Net architecture^17^. Moreover, we complement the architecture of the CNN by implementing a set of pixel-wise weights that account for microstructural features which are hard to identify in the tomographs and play a relevant role for the correct description of the 3D architecture of the alloy investigated.

## Material

The material under investigation is an AlSi12Cu3Ni2 alloy produced by gravity die casting provided by KS Kolbenschmidt GmbH. The microstructure of the alloy was investigated in two different samples taken from castings produced with different solidification rates (i.e. slower solidification rate for the training sample). Thus, the microstructures of the two samples are expected to vary with respect to the different solidification rates during casting. In our work, the microstructure will be characterised in terms of volume fraction of phases (aluminides, silicon and the α-Al matrix) as well as the global interconnectivity and local connectivity of each phase and of the hybrid network. The local connectivity is estimated by the topological parameter Euler number, *E*, which allows quantifying the number of connecting branches within a network^18^.

Cylindrical specimens with a diameter of 0.6 mm and a length of 2 mm were machined for synchrotron X-ray computed tomography (CT) scans. The experiments were carried out at the Helmholtz-Zentrum Geesthacht operated beamline P05 of the synchrotron source PETRA III at DESY, Hamburg^19^ using a voxel size of (1.2 µm³) and a volumetric field of view of 1833 × 1833 × 1833 µm³. The phase contrast provided by the synchrotron radiation at P05 is necessary to reveal simultaneously the Al and Si phases owing to their similar X-ray attenuations. Further experimental details can be found in^20^.

## Methodology

### Generation of ground truth data

For accurate training of a CNN, it is imperative to choose a suitable training dataset, i.e. a labelled ground truth which is sufficiently free from errors. To this purpose, we chose the reconstructed tomographic dataset of the AlSi12Cu3Ni2 sample with slower solidification rate. The microstructural constituents of this dataset were segmented following a method developed by the authors that combines data acquired using laboratory X-ray CT, synchrotron CT, chemical deep etching of the Al matrix and grey value thresholding^20^. This method reduces drastically the segmentation limitations for this kind of multiphase alloys, i.e. the very difficult segmentation of silicon and of the interfaces between aluminides and silicon. As a result, a realistic description of the 3D architecture of the alloys is obtained, particularly regarding interconnectivity of phases, which is crucial to understand their damage tolerance^13,14^. However, this methodology is time consuming, experimentally complex, and implies the destruction of the sample by chemical deep etching. As a reference, a working time of a few hundred hours must be considered from data acquisition to final image segmentation. This limitation hinders its serial applicability or its use for investigating the same sample at different conditions, e.g. before and after heat treatment. On the other hand, its high accuracy gives a suitable dataset that was used as ground truth for training the CNN applied in this work. The final objective is to segment any other tomographic datasets of this type of alloys in a fraction of the time needed with the method used to obtain the ground truth dataset. A segmented volume of 319 × 379 × 1000 voxels was used for training and validation of the CNN.

**Figure 1:**
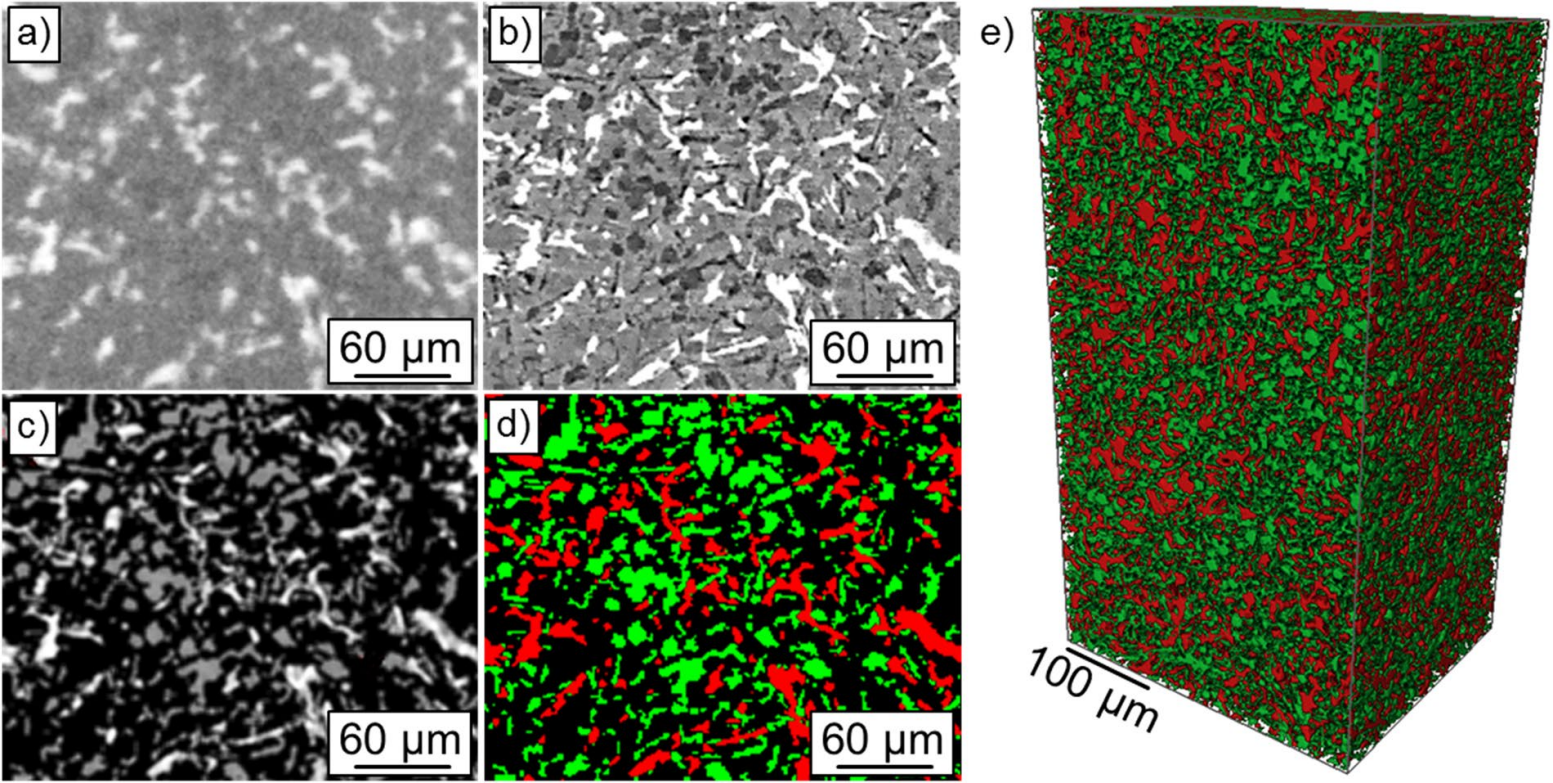
Ground truth dataset. 2D microstructure of the AlSi12Cu3Ni2 alloy acquired by (**a**) laboratory X-ray CT, (**b**) synchrotron CT and (**c**) synchrotron CT after deep etching of the Al matrix. In (**d**), a segmented slice shows Si (green), aluminides (red) and Al matrix (black). (**e**) 3D visualization of segmented aluminides and silicon.

Figure 1 shows reconstructed tomographic slices of the microstructure of the specimen used for training, acquired by (a) laboratory X-ray CT, (b) synchrotron CT and (c) synchrotron CT after chemical deep etching of the Al matrix. A combination of these three datasets allowed for the, until now, most reliable segmentation of all microstructural constituents, i.e. Al matrix, primary and eutectic Si (green) as well as aluminides (red) over a large representative volume (see Fig. 1(d,e)), as reported in^20^. The laboratory X-ray CT dataset reveals clearly the aluminides (bright phase in Fig. 1a). Using synchrotron CT, the Si and Al phase can be revealed separately, although phase contrast artefacts at aluminide/Al-matrix interfaces occur and can be misinterpreted as Si particles. Finally, the synchrotron CT dataset of the deep etched sample allows avoiding this effect and makes a more realistic segmentation of Si and interfaces between Si and aluminides possible. The Supplementary Video provides 3D visualizations of all datasets.

### Data augmentation and data splitting

The unsegmented and segmented ground truth datasets, comprising a stack with 1000 slices (16 bit grey scale before segmentation) were split into two parts:

1. The training dataset. This dataset is responsible for the performance of the CNN model. A total of 595 slices (slice no. 404–998) were chosen for training. A gap of 101 unused slices (301–401) between the test and training datasets ensures larger differences between the microstructural features of both datasets.
2. The validation/test dataset. The first 300 slices were used for testing the CNN after training. A section with a size of 256 × 256 pixels was cropped from the centre of each slice, i.e. resulting in a test dataset with a size of 256 × 256 × 300 voxels.

Augmentation of the training dataset was carried out to enhance generalization of the trained CNN. To this purpose, each slice was cropped from each corner into four overlapping images with a size of 256 × 256 pixels. Furthermore, rotations of 0°, 90° and 270° were applied to each cropped image. Moreover, brightness and contrast variations were performed randomly four times for each cropped slice in a range of $\pm 30 \%$ and $\{-20,+\,30\,\} \%$ with respect to the original images. Furthermore, a blurring (Gaussian) filter with a standard deviation for the Gaussian kernel of 0.0–1.0 and also random noise (i.e. adding a noise image with values $0\pm \,500$) was applied to some images to make the network even applicable to low quality reconstructions. All the augmentation operations improved the performance of the network regarding unseen test data. The size of the augmented training dataset was:

Augmented training dataset = 595 slices × 4 translations × 3 rotations × 4 grey scale transformations = 28,560 images.

Usually, several channels per image are used for training convolutional neural networks (e.g. one image for each of the three channels in the RGB colour space)^21^. In our case we used a configuration with a five-channel input. Instead of using colour channels of the same image for each input, five consecutive grey-scale slices from the tomographic reconstruction were used, namely the slice to be predicted (channel 3) as well as two slices before and two slices after the prediction slice. Thereby, we feed the CNN with 3D information, which may become relevant for the semantic segmentation.

### Implementation of the convolutional neural network

**Figure 2:**
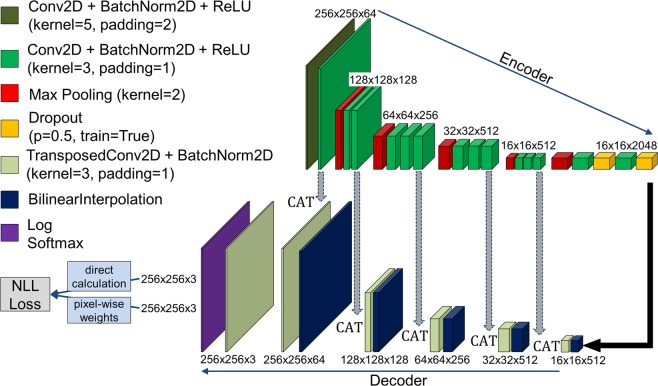
Encoder-decoder architecture of the used CNN based on^8,17^.

The PyTorch framework^22^ was used for the implementation of the CNN. This framework provides a user-friendly library of deep learning algorithms integrated in Python. Figure 2 shows the encoder-decoder architecture of the neural network. The architecture is a typical U-Net configuration^17^ with a handful of changes that were achieved by trial-and-error and were inspired mostly by the work of Azimi *et al*.^8^. We use a colour-scheme similar to the one given by Azimi *et al*. for better comparability. U-Net is a state of the art CNN for image segmentation tasks (e.g.^5^). The network was trained from scratch, as no pre-trained CNN seemed applicable for our application.

The CNN architecture consists of several layers that are structured in similar blocks with respect to the encoder or decoder part of the CNN. The first layer (olive) is a 2D convolution layer (Conv2D) with a kernel size of 5 × 5 pixels and padding 2. It has a 5 channel input (5 slices in our case, as explained above) and a 64 channel output. All other convolution layers (green) have a kernel size of 3 × 3 pixels and padding 1. Each convolution layer is followed by a batch normalization layer (BatchNorm2D) and a rectified linear unit (ReLU) function layer which is meant to add non-linearity between the convolutions^23^. The pooling layers (red) are max pooling layers. For the dropout (yellow) a probability of 0.33 was chosen. In the last encoder step, the image size is 16 × 16 pixels with 2048 channels. The decoder consists of two different types of layers: (1) the up-sampling layer, which is a bilinear interpolation in order to double the size of the image. (2) the transposed convolution layer (TransposedConv2D), which has again a kernel size of 3 × 3 pixels and padding 1. The convolution layer is followed by a batch normalization. At each stage of the decoder, the output of identical dimensions of the encoder and decoder are concatenated (CAT) to the new input for the next stage. The training was performed for 100 epochs using a batch size of 25 images. The dropout layers are omitted during testing. The Adam optimizer was used as it is implemented in PyTorch with an initial learning rate = 0.003, amsgrad = True and weight decay = 1.0E-4. This stochastic, gradient-based optimization algorithm was introduced in ref. ^24^.

### Pixel-wise weighted loss function

The widely used cross entropy loss function, which is defined in PyTorch as a series of the logarithmic Softmax and the 2D NLL Loss functions, was used during CNN training^25^. Besides this, we modified this loss function to a pixel-wise level in order to specify two features of the microstructures that are hard to identify in the tomographs and play a relevant role for the correct description of the 3D architecture of this type of alloys: (i) Si particles, which are revealed by phase contrast in the synchrotron tomography images and can, therefore, present a higher contrast at their borders than in their interior with respect to the Al matrix^3^, (ii) interfaces between aluminides and Si, that can be hard to identify owing to phase contrast artefacts^20^. Thus, the LogSoftmax function is calculated separately during CNN training while the NLL Loss function is weighted on a pixel-wise level before it is summed over the 2D image to a scalar overall error. The PyTorch implementation was derived analogously to the coding example given in^26^. A similar procedure is usually applied to weight classes which are under-represented in the training data set (class-balancing)^27^.

**Figure 3:**
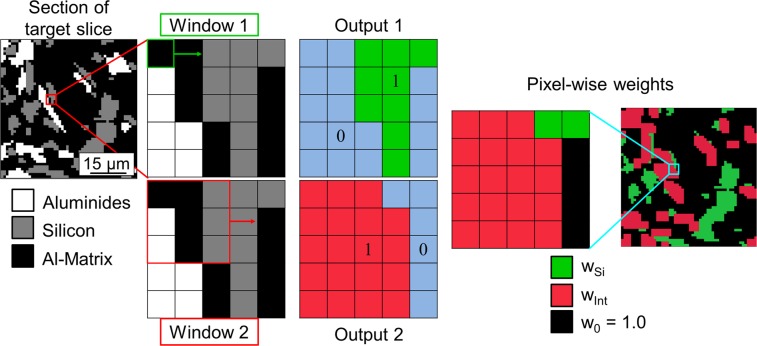
Generation of pixel-wise weights for each slice for training the CNN. The loss is weighted on a pixel-wise level to the purpose of highlighting the interfaces (**ω**_**Int**_) and silicon (**ω**_**Si**_) pixels during training.

Figure 3 describes the procedure used to generate the pixel-wise weights for each target image.

1. The target image is duplicated two times.
2. A window with a size of one pixel (Window 1 in Fig. 3) is slided over one of the duplicate images, setting each pixel that is labelled in the target as silicon to a value of 1 and the rest to 0. Output 1 in Fig. 3 shows the results of this step in a small region of the target image. The pixels turned into value = 1 are shown in green and those turned into value = 0 in blue for better visualization. The resulting image is analogous to the target Si phase.
3. A window with a size of 3 pixels (Window 2 in Fig. 3) is slided over the other duplicate image, setting all the pixels contained in the window to a value = 1 if at least one pixel of class “aluminides” and one pixel of class “silicon” is contained within the window. If this condition is not satisfied, the pixels within the 3 × 3 pixels window are set to value = 0 unless they have already been transformed to value = 1 in a previous position of the sliding window. Output 2 in Fig. 3 shows the results of this step in a small region of the target image. The pixels turned into value = 1 are shown in red and those turned into value = 0 in blue for better visualization. The purpose of this transformation is to obtain a map that contains extended Si-aluminide interfaces.
4. The two outputs (Output 1 and Output 2 in Fig. 3) are combined to obtain the final pixel-wise weight image. The resulting weights in the final image are defined for each pixel in the following way:

- If a pixel has value = 0 in Output 1 and value = 1 in Output 2 then the weighting factor ${\omega }_{{\rm{Int}}}$ will be applied to this pixel.
- If a pixel has value = 1 in Output 1 and 0 in Output 2 then the weighting factor ${\omega }_{{\rm{Si}}}$ will be applied to this pixel.
- Each pixel with value = 1 in Output 1 and in Output 2 will be weighted as a possible interface pixel using the factor ${\omega }_{{\rm{Int}}}$.
- The weighting ${\omega }_{0}$ = 1.0 is the weighting factor for each pixel with value = 0 in both the Output 1 and Output 2 images.

The pixel-wise weights must be defined before training the CNN since they are not trainable parameters as the term “weights” is usually understood in the context of artificial neural networks. We applied three different pixel-wise weight configurations:

1. ***ω***_0_ = ***ω***_Si_ = ***ω***_Int_ = 1.0; this represents the case for which no pixel-wise weights are applied.
2. ***ω***_0_ = 1.0, ***ω***_Si_ = 1.15 and ***ω***_Int_ = 1.2.
3. ***ω***_0_ = 1.0, ***ω***_Si_ = 1.25 and ***ω***_Int_ = 1.5.

### Evaluation of the CNN

We chose four different parameters to evaluate the performance of the CNN.

1. The accuracy, *a*, in each slice, where: *a* = (Number of correctly classified pixels) ⁄ (256 × 256 pixels).
2. The difference in volume fraction of each phase with respect to the ground truth dataset.
3. The difference in global interconnectivity of each phase and of the hybrid network formed by Si and aluminides with respect to the ground truth dataset.
4. The difference in local connectivity of each phase and of the hybrid network, expressed by the Euler number, *E* (i.e. connecting branches within a network)^18^.

### Hardware and software

The training and testing of the CNN was performed on a Fujitsu Celsius R930Power Work Station with 12 × 16 GB DDR3 working memory and two Intel Xeon E5-2667v2 3.30 GHz 25MB Turbo Boost processors with 8 kernels each. The machine was equipped with an NVIDIA RTX 8000 GPU^28^ for GPU accelerated computations. The programming was done in Python3 using standard libraries for pre- and post-processing as well as PyTorch as machine learning framework. 3D image analysis was done with the software ImageJ and Aviso 9.5. The total training time of the CNN for 100 epochs (i.e. 100 epochs × 28.560 images/25 (images/batch) = 114240 iterations) was ~32 hours.

## Results and Discussion

**Figure 4:**
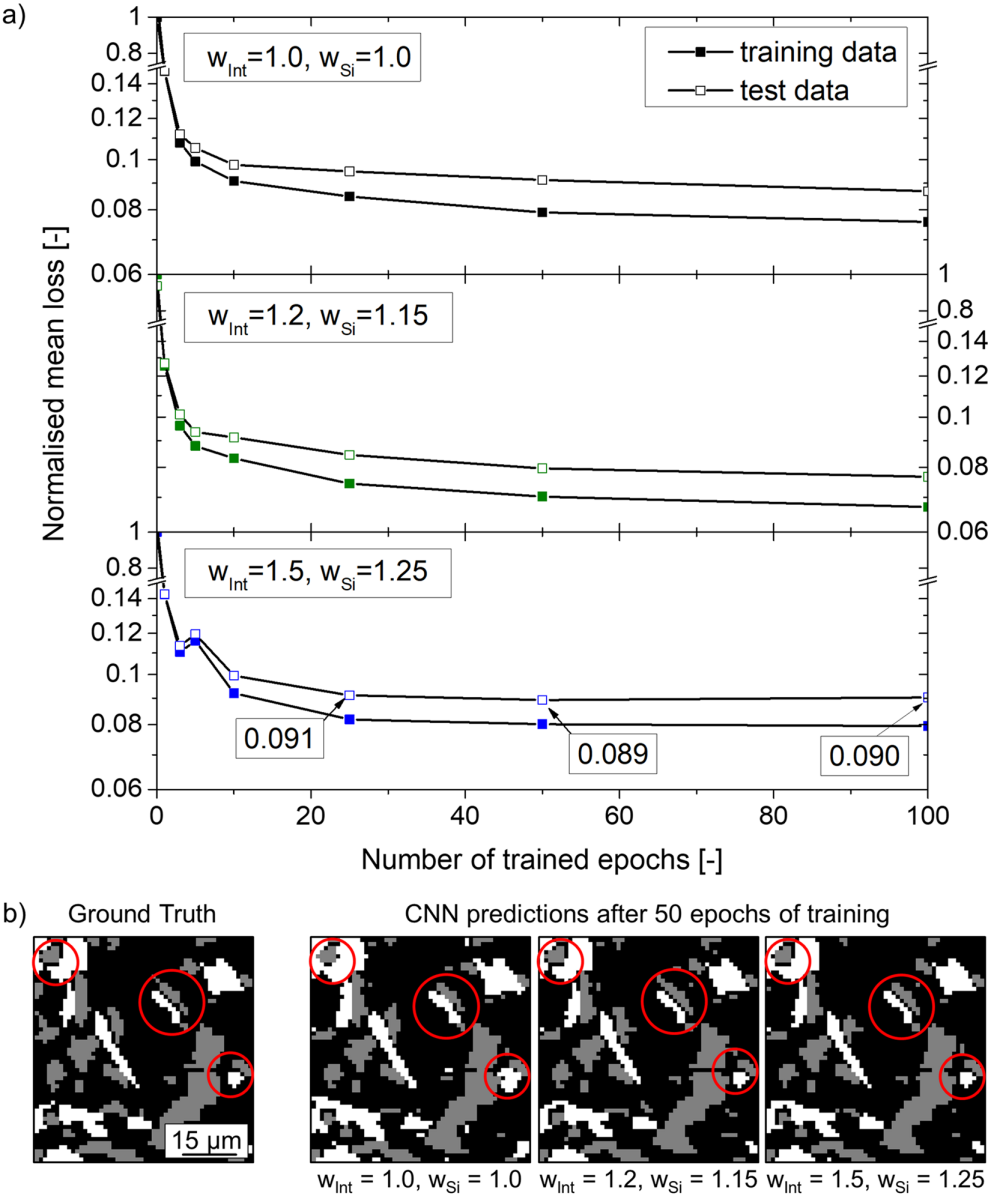
Normalised mean loss for the tested pixel-wise weight factors ω_Int_, ω_Si_ as a function of the trained epochs.

The normalised loss functions (i.e. a random segmentation after 0 epochs of training results in a loss of 1.0) are plotted in Fig. 4 as a function of the training epochs for the three pixel-wise weight configurations. The mean loss taking into account all slices in the training or test datasets are shown. It can be seen that >80% of convergence is reached after epoch 1. After 50 epochs, the slope of the loss function is close to zero and it even becomes slightly positive for the test data with high pixel-wise weights (bottom diagram in Fig. 4a).

A representative region of a slice segmented by the CNN at epoch 50 as well as the ground truth of the same area are shown in Fig. 4(b). The influence of the applied pixel-wise weights can be clearly appreciated qualitatively. While the Si-aluminide interfaces (see red circles) are wrongly estimated by the CNN for the case with pixel-wise weights = 1.0, these interfaces are segmented precisely when weights ≠ 1.0 are applied.

**Figure 5:**
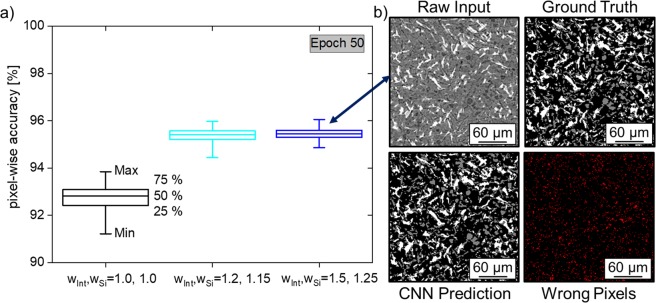
(**a**) Pixel-wise accuracy after 50 epochs of training. The minimum accuracy for the case of high pixel-wise weights does not fall below 94%. The CNN predicted image slice is very similar to its respective ground truth (**b**).

The accuracy of the segmentation, *a*, in the test data is shown in Fig. 5, for the three pixel-wise weight configurations at epoch 50. The accuracy is defined as the percentage of pixels correctly segmented with respect to the ground truth. The accuracy is the lowest if ω_Int_ and ω_Si_ = 1.0 (~93%), while it is the highest if ω_Int_ = 1.5 and ω_Si_ = 1.25 (~96%). A slice of the latter condition, i.e. ω_Int_ = 1.5 ω_Si_ = 1.25 is presented in Fig. 5(b) showing the raw input data, the ground truth segmentation, the CNN prediction and the difference between ground truth and prediction. From a qualitative point of view, this case with the highest accuracy in segmentation presents a quality that makes it difficult to tell whether the CNN or human segmentation is better.

**Figure 6:**
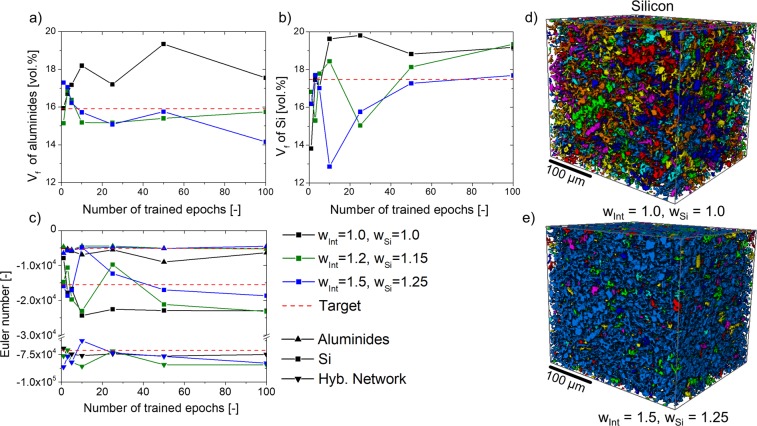
(**a**) Volume fraction of aluminides and (**b**) Si as well as (**c**) the Euler number of aluminides, Si and the hybrid network as a function of the trained epochs. In (**d**,**e**) Si particles are shown for the segmentation with and without the application of pixel-wise weights, respectively. The colours indicate individual particles.

The pixel-wise segmentation accuracy *a* is not necessarily the most relevant quality parameter in the context of materials science. Particularly, in the case of the alloys studied in this work, the volume fractions of the individual phases as well as their individual and combined 3D connectivity are crucial to understand their damage behaviour^13,14^. Therefore, the segmentation carried out by the CNN was compared to the target volume fraction of aluminides and Si as well as to the Euler number of Si, aluminides and the hybrid 3D network formed by them. The results obtained are shown as a function of the training epochs in Fig. 6(a–c). The global interconnectivity of Si, aluminides and the hybrid Si + aluminides networks was also computed and it is shown in the Supplementary Fig. 1.

The red dashed line indicates the ground truth values. The results clearly show that the application of pixel-wise weight factors increases the quality of the CNN segmentation. The best CNN prediction is given for ***ω***_0_ = 1.0, ***ω***_Si_ = 1.25 and ***ω***_Int_ = 1.5 at 50 epochs of training. For this case, the volume fractions of both phases as well as the Euler numbers of the phases and of the hybrid network are in very good agreement with the ground truth. The fact that 50 epochs of training give better results than 100 can be understood taking into account the slight increase in the loss function for later epochs (see description of Fig. 4 above), which indicates that some overfitting of the training is obtained beyond epoch 50 for the application of high pixel-wise weights. A 3D visualization of the network of primary and eutectic Si is shown in Fig. 6(d,e) for the CNN trained with pixel-wise weights ω_Int_ = ω_Si_ = 1.0 and ω_Int_ = 1.5, ω_Si_ = 1.25, respectively. The different colours indicate individual particles. It can be seen that the high interconnectivity of the network of Si is accurately segmented only if the pixel-wise weights ≠1.0 are applied during training, as it should be expected according to the ground truth segmentation (see Fig. 1e).

### Application of the trained CNN to a second volume with a different solidification rate during casting

The applicability of the trained CNN to segment microstructures different from those of the volume considered for training was investigated. To this purpose, a segmentation of a second tomographic volume of the same alloy but acquired by casting with a faster solidification rate was analysed. Therefore, we used the CNN trained with the application of pixel-wise weights ω_Int_ = 1.5, ω_Si_ = 1.25 and 50 epochs of training. The segmentation of a volume of 256 × 256 × 1925 voxels took less than 10 minutes.

**Figure 7:**
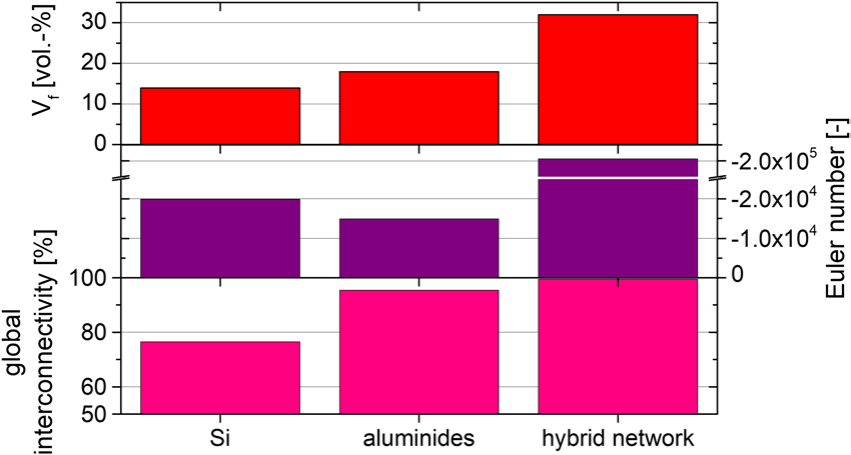
Quantitative evaluation of volume fraction, interconnectivity and Euler number for the AlSi12Cu3Ni2 alloy produced with a faster solidification rate during casting compared to the training sample.

The quantitative evaluation of the segmentation (shown in Fig. 7) reveals volume fractions of ~14 vol.%, 18 vol.% and ~32 vol.% for the Si-, aluminide- and the hybrid network, respectively.

The larger volume fraction of aluminides with respect to the alloy with slower solidification rate is a reasonable consequence, because the higher solidification rate may result in microsegregations that increase the fraction of aluminides. As a consequence, the relative volume fraction of Si must decrease, which is confirmed by the segmentation results. Moreover, the global interconnectivity of the aluminide and hybrid networks reaches ~95% and 99%, respectively, which is comparable to the ones achieved for the training sample. In contrast, the Si network from faster solidification shows ~15% less global interconnectivity compared to the training sample. The Euler number, i.e. the local connectivity, of all networks reveals more negative values as compared to the training microstructure, which is again in agreement with the fact that the alloy of the second sample experiences higher solidification rates. Thus, this results in a microstructure with smaller inter-particle distances, which in turn increase the potential for the formation of a larger amount of connecting branches within the networks^29^. Besides the fact that a completely automatic segmentation can save a substantial amount of human working time, the method provides the opportunity of a more user-independent segmentation technique and it is therefore more objective.

## Conclusions

We have shown that the training of a convolutional neural network is a very appropriate tool to objectively segment the complex 3D microstructure of cast Al-Si alloys revealed by synchrotron tomography. The application of a pixel-wise weighted loss function during training that enhances relevant microstructural features that are hard to detect in the tomographic images, i.e. the Si phase revealed by phase contrast and Si-aluminide interfaces, made it possible to achieve a precise segmentation of the tomographs. Training time of the CNN with a U-Net architecture and a human–based segmentation ground truth took a total time of ~16 hours for 50 epochs. Once the CNN is appropriately trained, the total operation time for the segmentation of a large volume (i.e. 256 × 256 × 1925 voxels) was reduced to <1% of the time needed with human-based segmentation.

## Supplementary information

Supplementary Information 2

Supplementary Information 3

## References

[1] Maire E, Withers PJ (2014). Quantitative X-ray tomography. International Materials Reviews.

[2] Wang C, Steiner U, Sepe A (2018). Synchrotron Big Data Science. Small.

[3] Baruchel J (2006). Advances in synchrotron radiation microtomography. Scripta Materialia.

[4] Liu X, Deng Z, Yang Y (2019). Recent progress in semantic image segmentation. Artificial Intelligence Review.

[5] Maier A, Syben C, Lasser T, Riess C (2019). A gentle introduction to deep learning in medical image processing. Zeitschrift Fur Medizinische Physik.

[6] Mou L, Bruzzone L, Zhu XX (2019). Learning spectral-spatialoral features via a recurrent convolutional neural network for change detection in multispectral imagery. In IEEE Transactions on Geoscience and Remote Sensing.

[7] Anwar SM (2018). Medical Image Analysis using Convolutional Neural Networks: A Review. Journal of Medical Systems.

[8] Azimi SM, Britz D, Engstler M, Fritz M, Mücklich F (2018). Advanced steel microstructural classification by deep learning methods. Scientific Reports.

[9] Gola J (2018). Advanced microstructure classification by data mining methods. Computational Materials Science.

[10] Iglesias JCÁ, Santos RBM, Paciornik S (2019). Deep learning discrimination of quartz and resin in optical microscopy images of minerals. Minerals Engineering.

[11] Kaira CS (2018). Automated correlative segmentation of large Transmission X-ray Microscopy (TXM) tomograms using deep learning. Materials Characterization.

[12] Asghar Z, Requena G, Boller E (2011). Three-dimensional rigid multiphase networks providing high-temperature strength to cast AlSi10Cu5Ni1-2 piston alloys. Acta Materialia.

[13] Bugelnig K (2018). Influence of 3D connectivity of rigid phases on damage evolution during tensile deformation of an AlSi12Cu4Ni2 piston alloy. Materials Science and Engineering A.

[14] Bugelnig K (2018). Revealing the Effect of Local Connectivity of Rigid Phases during Deformation at High Temperature of Cast AlSi12Cu4Ni(2,3)Mg Alloys. Materials.

[15] Kruglova A (2018). 3D connectivity of eutectic Si as a key property defining strength of Al–Si alloys. Comp. Mater. Sci..

[16] Requena G (2011). The Effect of the Connectivity of Rigid Phases on Strength of Al-Si Alloys. Adv. Eng. Mater..

[17] Ronneberger, O., Fischer, P. & Brox, T. U-Net: Convolutional Networks for Biomedical Image Segmentation. *Medical Image Computing and Computer-Assisted Intervention (MICCAI)*, 234–241 (2015).

[18] Toriwaki J, Yonekura T (2002). Euler Number and Connectivity Indexes of a Three Dimensional Digital Picture. Forma.

[19] Wilde, F. *et al*. MicroCT at the imaging beamline P05 at PETRA III. In *AIP Conference Proceedings* 1741. 030035-1 (2016).

[20] Bugelnig K (2018). Optimized Segmentation of the 3D Microstructure in Cast Al-Si Piston Alloys. Practical Metallography.

[21] Gowda Shreyank N., Yuan Chun (2019). ColorNet: Investigating the Importance of Color Spaces for Image Classification. Computer Vision – ACCV 2018.

[22] “PyTorch” [Online]. Available: https://www.pytorch.org. [Accessed 17 09 2019].

[23] Jung, W. *et al*. Restructuring Batch Normalization to Accelerate CNN Training. Retrieved from, https://arXiv:1807.01702v2 (2018).

[24] Kingma, D. P. & Lei, B. J. Adam: A Method For Stochastic Optimization. In *proceedings at* ICLR arXiv:1412.6980v9 (2015).

[25] Subramanian, V. Deep learning with PyTorch: a practical approach to building neural network models using PyTorch. Birmingham, *Packt Publishing* (2018).

[26] https://discuss.pytorch.org/t/weighted-pixelwise-nllloss2d/7766. [Accessed 23 09 2019].

[27] Johnson, J. M. & Khoshgoftaar, T. M. Survey on deep learning with class imbalance. *Journal of Big Data*, **6**(27), 10.1186/s40537-019-0192-5 (2019).

[28] NVIDIA QUADRO RTX 8000 datasheet [online]. Available, https://www.nvidia.com/en-us/design-visualization/quadro/rtx-8000/ [Accessed 17 09 2019].

[29] Zamani M, Seifeddine S, Azuzuderourei M (2013). The Role of Sr on Microstructure Formation and Mechanical Properties of Al-Si-Cu-Mg Cast Alloy. In Light Metals.

