# Supplementary material for: Semantic segmentation of synchrotron tomography of multiphase Al-Si alloys using a convolutional neural network with a pixel-wise weighted loss function
Source: Sci Rep. 2019 Dec 23;9:19611. doi: 10.1038/s41598-019-56008-7 (PMC6928157; doi:10.1038/s41598-019-56008-7)
Supplement: Supplementary file 1 — Supplementary Information 2 [file 41598_2019_56008_MOESM1_ESM.pdf]

Semantic segmentation of synchrotron tomography of multiphase Al-Si alloys using a convolutional neural network with a pixel-wise weighted loss function

Tobias STROHMANN*[1], Katrin BUGELNIG[1], Eric BREITBARTH[1], Fabian WILDE [2], Thomas STEFFENS[3], Holger GERMANN[3],

Guillermo REQUENA[1,4]

Supplementary figures

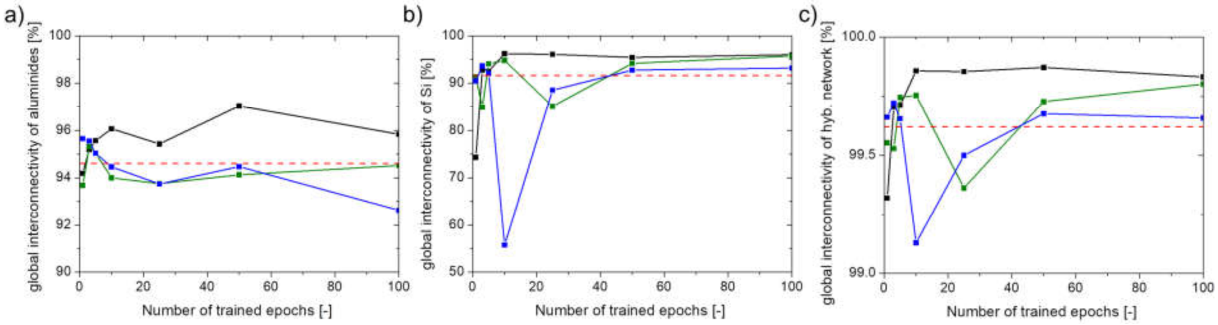

Supplementary figure 1 - Global interconnectivity of Si, aluminides and the hybrid Si+aluminides networks
